# Post-traumatic stress disorder and childhood emotional abuse are markers of subthreshold bipolarity and worse treatment outcome in major depressive disorder

**DOI:** 10.1097/YIC.0000000000000380

**Published:** 2021-10-20

**Authors:** Paolo Olgiati, Alessandro Serretti

**Affiliations:** Department of Biomedical and Neuromotor Sciences, University of Bologna, Bologna, Italy

**Keywords:** bipolar spectrum, childhood maltreatment, hypomanic switch, mixed depression, post-traumatic stress disorder

## Abstract

Post-traumatic stress disorder (PTSD) and childhood maltreatment (CMT: parental neglect; emotional, physical and sexual abuse) have been linked to bipolar disorder but they are also common in major depressive disorder (MDD). Our objective was to investigate their association with the bipolar spectrum and antidepressant treatment outcome in 482 outpatients with DSM-IV MDD treated in the Combining Medications to Enhance Depression Outcomes trial for 28 weeks Bipolar spectrum score included age of onset <21 years, subthreshold hypomania (a period of elated or irritable mood with at least two concurrent hypomanic symptoms, which did not fulfill DSM criteria for hypomanic/manic episode) and depressive mixed state (DMX). PTSD subjects (*n* = 107; 22%) had more severe depression (*P* < 0.0001), work and social impairment (*P* = 0.0031), comorbid anxiety disorders (*P* < 0.0001) and increased suicidality (*P* = 0.0003). Bipolar spectrum score was higher with PTSD comorbidity (*P* = 0.0063) and childhood emotional abuse (*P* = 0.0001). PTSD comorbidity was associated with residual suicidality (*P* = 0.0218) after 6 weeks of antidepressant use whereas childhood emotional abuse [odds ratio (OR), 1.01–2.22], subthreshold hypomania (OR, 1.04–4.09) and DMX (OR, 1.00–4.19) were predictors of mood switch. These results corroborate the role of PTSD and childhood emotional abuse as markers of bipolar spectrum and prognostic factors during antidepressant treatment.

## Introduction

On the basis of current diagnostic criteria, bipolar disorders are only identifiable by ascertaining manic or hypomanic episodes. This approach is not devoid of weaknesses (Nusslock and Frank, 2011). A first concern is that in bipolar patients the onset of mood activation is often preceded by a large number of depressive episodes, thus there is a lengthy time period from the point of illness onset to correct diagnosis. Second, milder, albeit clinically significant, bipolar spectrum syndromes would be included, by default, in major depressive disorder (MDD), with detrimental effects on illness course (e.g. manic switch and rapid cycling) related to inappropriate treatment. In the last few decades, a great research effort has allowed identifying reliable markers of bipolarity such as mixed depression [i.e. a major depressive episode (MDE) with few concurrent hypomanic symptoms] (Akiskal *et al.*, 2005; Benazzi, 2005; Perugi *et al.*, 2015) and subthreshold hypomanic episodes occurring outside depressive phases (Angst *et al.*, 2003; Zimmermann *et al.*, 2009; Serretti *et al*., 2021). Notwithstanding this progress, whenever information on prior manic or hypomanic episodes is not available, the correct identification of bipolar depression remains a challenge. In this context, post-traumatic stress disorder (PTSD) might deserve attention as a marker of bipolar spectrum. PTSD comorbidity involves up to one-third of patients with major depression (Green *et al.*, 2006; Campbell *et al.*, 2007) but several lines of evidence support its connection with bipolar disorder. For instance, it is known that individuals who have been exposed to traumatic experiences or complicated grief and exhibit PTSD manifestations are at increased risk of developing hypomanic symptoms (Dell’Osso *et al.*, 2012). PTSD is actually one of the most frequent diagnoses in patients with bipolar disorder (Otto *et al.*, 2004; Goldberg and Garno, 2005; Neria *et al.*, 2008; Assion *et al.*, 2009) and vice-versa (Hernandez *et al.*, 2013; McLay *et al.*, 2014). Moreover, the likelihood of PTSD in bipolar subjects is 4–5 times higher relative to patients with MDD (Dilsaver et al., 2007, 2008). Childhood maltreatment (CMT) – which includes parental neglect, emotional abuse, physical abuse and sexual abuse – is even more related to bipolar illness. In a meta-analysis individuals with bipolar disorder were 2.6 times more likely to report CMT compared to non-clinical controls (Palmier-Claus *et al.*, 2016). Additionally, among bipolar subjects, those who had been exposed to CMT were more likely to experience their first episode earlier (Larsson *et al.*, 2013) as well as to develop rapid cycling and suicidal behavior (Garno *et al.*, 2005; Etain *et al.*, 2013; Aas *et al.*, 2014). CMT has also been linked to suicidality. In the most comprehensive review, which analyzed over 260 000 individuals from 68 studies, CMT was associated with two-fold to three-fold increased risks for suicide ideation and attempts (Angelakis *et al*., 2019).

The aim of this study was to investigate the diagnostic and prognostic roles of PTSD and CMT in MDD, to disentangle their association with bipolar spectrum, the likelihood of mood activation and the persistence of suicidal tendency during antidepressant use.

## Methods

### Sample

This study was a secondary analysis of the Combining Medications to Enhance Depression Outcomes (CO-MED) trial, which was carried out at six primary care sites and nine psychiatric care centers across the USA (Rush *et al.*, 2011). Eligible subjects were of age 18–75 years, with DSM IV-based MDD and HDRS_17_ scores ≥16. Exclusion criteria were psychotic depression or bipolar (DSM IV) illness and admission to psychiatric inpatient facilities. The CO-MED trial enrolled 665 subjects from March 2008 to September 2009. Our analysis involved 482 participants recruited until February 2009.

### Ethical issue and informed consent

The CO-MED trial was conducted according to the Principles of Helsinki Declaration and its protocol was reviewed and approved by ethical committees at local recruitment sites (Rush *et al.*, 2011). All subjects selected by clinicians were included in the screening phase after obtaining their written informed consent. This research group certifies that data collected for the CO-MED trial were exclusively used for scientific investigation. Before obtaining access to data, the objectives of our investigation were clearly described in the request form (Serretti *et al*., 2021).

### Treatments

CO-MED was designed as a single-blind (participant only), placebo-controlled trial in which eligible subjects were randomly assigned to one of the following treatment arms: (1) escitalopram plus placebo; (2) bupropion SR plus escitalopram and (3) venlafaxine XR plus mirtazapine. The trial included a short-term (12 weeks) treatment followed by a continuation phase (weeks 12–28) (Rush *et al.*, 2011).

### Assessment

Sociodemographic characteristics were collected by means of a specific form including age, gender, ethnic group, education and monthly income (Rush *et al.*, 2011). The Mini-International Neuropsychiatric Interview (M.I.N.I) (Sheehan *et al.*, 1998) was used to validate the diagnosis of MDD and exclude psychotic and bipolar illness, to assess some clinical features such as chronic or recurrent depression, the number of past depressive episodes and age at onset of the first episode and to ascertain the lifetime occurrence of subthreshold hypomanic episodes (see below). Depressive episode was thoroughly assessed by administering the 30-item Inventory of Depressive Symptomatology-Clinician Rating (IDS-C_30_) (Corruble *et al.*, 1999) and the 16-item Quick Inventory of Depressive Symptomatology (QIDS-C_16_) (Rush *et al.*, 2003), the Concise Associated Symptoms Tracking (CAST) (Trivedi *et al.*, 2011a) and Concise Health Risk Tracking (CHRT) (Trivedi *et al.*, 2011b) scales, which respectively, assessed irritability and suicide propensity and ideation, the Altman Self-Rating Mania Scale (ASRM) (Altman, 1998) to ascertain intra-MDE hypomanic symptoms and the Work and Social Adjustment Scale (WSAS) (Mundt *et al.*, 2002) to ascertain functional impairment. The individual assessment was completed by the Psychiatric Diagnostic Screening Questionnaire (PDSQ) (Zimmerman and Mattia, 1999), which investigated comorbid PTSD (post-traumatic scale ≥8) and anxiety disorders, and a questionnaire that was specifically developed to explore CMT subtypes (Medeiros *et al.*, 2021).

### Bipolar validators and bipolar spectrum score

The following variables were included among bipolar illness validators: (1) age at onset of first mood disorder episode (<21 years) (Benazzi, 2009); (2) per year recurrence of mood disorder episodes (Mazzarini *et al.*, 2018); (3) lifetime occurrence of subthreshold hypomania: a period of elated or irritable mood with at least two concurrent hypomanic symptoms (MINI interview), which did not fulfill DSM criteria for hypomanic/manic episode (Angst *et al.*, 2003; Serretti *et al*., 2021); (4) depressive mixed state (DMX) (Benazzi, 2001, 2008): an MDE with three or more hypomanic symptoms (ASMR), assessed before antidepressant treatment start. Subsequently, bipolar spectrum score was calculated as follows: A. age of onset <21 years: 1 point; + B. lifetime occurrence of subthreshold hypomania: 2 points; + C. DMX: 2 points.

### Antidepressant treatment outcome

In prior CO-MED analysis, Medeiros and colleagues (2021) investigated the impact of CMT on antidepressant-treatment outcomes. Here, instead, we focused on PTSD comorbidity and analyzed its association with response (>50% decrease in QIDS score from baseline) and remission (QIDS≤5) after six weeks of antidepressant use. In addition, we analyzed the association of PTSD and CMT with residual levels of suicide propensity and ideation (CHRT) as well as with mood activation (ASRM score ≥6) (Altman, 1998) occurring after ≥14 days of antidepressant use.

### Statistical analysis

Univariate analyses were performed using Student’s *t* and Chi-square tests for continuous and categorical variables respectively; due to a large number of comparisons, the statistical significance threshold was conservatively set at alpha = 0.025. Multivariate analysis was conducted by means of multiple regression and multiple logistic regression (MLR) analysis including variables with *P* ≤ 0.10 at the univariate level. Statistical software was OpenStat version 8 December 2014 (https://openstat.info/OpenStatMain.htm).

## Results

Sample’s characteristics are summarized as follows: age: 43.15 ± 12.46 years; males: 144 (30%); depression severity (IDS-C_30_): 38.63 ± 9.13. PTSD and CMT were reported by 107 (22%) and 260 (54%) patients, respectively. Subjects with PTSD endorsed a larger number of CMT events than their counterpart without PTSD (PTSD: 1.92 ± 1.56; no PTSD: 1.13 ± 1.35; *t* = 4.71; *P* < 0.0001): in fact, they were more often victims of parental neglect (PTSD: 62/107; no PTSD: 132/375; χ^2^ = 17.91; *P* < 0.0001), childhood emotional abuse (PTSD: 68/107; no PTSD: 144/375; χ^2^ = 21.37; *P* < 0.0001) and childhood physical abuse (PTSD: 42/107; no PTSD: 67/375; χ^2^ = 21.75; *P* < 0.0001). Nevertheless, from the multivariate analysis of CMT subtypes, physical abuse was the only independent predictor of PTSD comorbidity (MLR: χ^2^ = 27.26; *P* < 0.0001: OR, 1.89; 95% CI, 1.04– 3.83).

### Post-traumatic stress disorder comorbidity and depression severity

Comparisons between PTSD and no PTSD groups are displayed in Table 1. Subjects with PTSD were characterized by higher depression scores at baseline (IDS-C_30_) (*t* = 4.10; *P* < 0.00001), greater work and social impairment (*t* = 2.97; *P* = 0.0031), increased suicidality (CHRT suicide propensity scale: *t* = 3.63; *P* = 0.0003; CHRT suicide risk scale: *t* = 2.99; *P* = 0.0029) and more anxiety disorder comorbidity (panic disorder: *t* = 9.69; *P* < 0.00001; generalized anxiety: *t* = 8.04; *P* < 0.00001; obsessive compulsive disorder (OCD): *t* = 8.03; *P* < 0.00001; social phobia: *t* = 5.86; *P* < 0.0001) (Table 1). Their symptom profile included higher levels of negative self-outlook (*t* = 3.08; *P* = 0.0022), anxious mood (*t* = 3.15; *P* = 0.0017), difficulty in falling asleep (*t* = 4.26; *P* < 0.0001), middle nocturnal insomnia (*t* = 3.29; *P* = 0.0012) and poor concentration (*t* = 2.71; *P* = 0.0070). MLR analysis identified seven independent predictors of PTSD comorbidity: overall depression score (OR, 0.89– 0.98), negative self-outlook (OR, 1.01–1.96), difficulty in falling asleep (OR, 1.00–1.61), middle nocturnal insomnia (OR, 1.01–1.77) and comorbid panic disorder (OR, 1.07–1.23), generalized anxiety (OR, 1.07–1.29) and OCD (OR, 1.06–1.47).

**Table 1:** Patients with and without post-traumatic stress disorder

	With PTSD (*N* = 107)	Without PTSD (*N* = 375)	*P*
Age	42.2 ± 11.2	43.4 ± 12.8	0.3902
Gender (male; %)	33 (0.30)	112 (0.29)	0.9990
Ethnic group (Caucasian; %)	67 (0.63)	254 (0.68)	0.3830
Education (years)	13.4 ± 2.5	13.8 ± 3.2	0.1346
Depression (IDS-C_30_)			
Baseline	41.8 ± 9.8	37.7 ± 8.7	<0.0001*
Week 6	23.5 ± 12.6	20.1 ± 11.4	0.0187*
Work/social impairment	29.3 ± 7.9	26.4 ± 8.9	0.0031*
Comorbidity (PDSQ)			
Panic disorder	8.6 ± 5.2	3.3 ± 4.1	<0.0001*
GAD	8.6 ± 1.8	6.0 ± 3.2	<0.0001*
OCD	2.2 ± 2.3	0.8 ± 1.4	<0.0001*
Social phobia	7.8 ± 4.7	4.7 ± 4.9	<0.0001*
Suicidal risk (CHRT)			
Propensity	18.7 ± 8.1	15.5 ± 8.2	0.0003*
Ideation	2.7 ± 3.0	1.9 ± 2.4	0.0089*
Depression symptoms (IDS-C_30_)			
Negative self-outlook	2.1 ± 0.9	1.8 ± 0.9	0.0022*
Anxious mood	2.0 ± 0.7	1.7 ± 0.8	0.0017*
Difficulty in falling asleep	2.3 ± 1.0	1.8 ± 1.3	<0.0001*
Middle nocturnal insomnia	2.4 ± 1.0	2.0 ± 1.1	0.0012*
Poor concentration	1.9 ± 0.7	1.7 ± 0.8	0.0070*
*Significant *P* < 0.01. ^a^Multiple logistic regression analysis: Chi-square = 85.09; df = 13; *P* < 0.0001 GAD, generalised anxiety; OCD, obsessive compulsive disorder.			
Predictors	OR (95% CI)		
Panic disorder	1.15 (1.07–1.23)		
GAD	1.22 (1.07–1.29)		
OCD	1.24 (1.06–1.47)		
Baseline IDS-C_30_	0.93 (0.89–0.98)		
Negative self-outlook	1.41 (1.01–1.96)		
Difficulty in falling asleep	1.27 (1.00–1.61)		
Middle nocturnal insomnia	1.34 (1.01–1.77)		
Sociodemographic characteristics and depression features. ^a^Only statistically significant predictors are shown. CHRT, Concise Health Risk Tracking; GAD, generalised anxiety; IDS-C_30_, 30-item Inventory of Depressive Symptomatology-Clinician Rating; OCD, obsessive compulsive disorder; PTSD, post-traumatic stress disorder; PDSQ, Psychiatric Diagnostic Screening Questionnaire.			

### Post-traumatic stress disorder comorbidity and bipolar features

The mean age at depression onset was 23.35 ± 13.50 years but in 262 subjects (54%) the first episode occurred before 21 years of age. 68 subjects (14%) met the criteria for DMX and 48 (10%) for subthreshold hypomania. Comparisons of bipolar features by PTSD classifier are displayed in Table 2. The PTSD group was found to differ from individuals without PTSD in terms of younger age at depression onset (*t* = 2.48; *P* = 0.0136), greater lifetime presence of subthreshold hypomania (Chi-square = 5.39; *P* = 0.020) and higher bipolar spectrum score (*t* = 2.77; *P* = 0.0063). Conversely, the distribution of DMX was not statistically different between the two groups. Among bipolar spectrum symptoms, irritability (CAST: *t* = 3.86; *P* = 0.0002), increased talkativeness (*t* = 2.86; *P* = 0.005) and reduced need for sleep (*t* = 2.67; *P* = 0.0084) showed the strongest association with PTSD. By performing MLR analysis, bipolar spectrum score emerged as an independent predictor of PTSD (OR, 1.05–1.54) along with irritability (OR, 1.14–3.76), difficulty in falling asleep (OR, 1.03–1.54) and middle nocturnal insomnia (OR, 1.02–1.61) (Table 2). The association between bipolar spectrum score and PTSD was no more significant (OR, 0.99–1.48) when CMT subtypes were added to other variables; thus, the new PTSD predictors became irritability (OR, 1.12–3.81), difficulty in falling asleep (OR, 1.02–1.55), middle nocturnal insomnia (OR, 1.01–1.61) and childhood physical abuse (OR, 1.05–3.63) (Table 2).

**Table 2:** Post-traumatic stress disorder correlates: bipolar features, irritability, insomnia and childhood maltreatment

	With PTSD (*N* = 107)	Without PTSD (*N* = 375)	*P*
Age of depression onset	20.5 ± 13.6	24.2 ± 13.4	0.0136*
N. episodes/illness years	0.5 ± 1.2	0.3 ± 0.6	0.1186
Depressive mixed state (DMX)	20 (0.19)	45 (0.12)	0.0742
Subthreshold hypomania	17 (0.16)	31 (0.08)	0.0204*
Bipolar spectrum score (0–5)	1.3 ± 1.2	0.9 ± 1.0	0.0063*
Irritability (CAST)	0.9 ± 0.4	0.7 ± 0.5	0.0002*
Increased talkativeness (ASRM)	0.7 ± 1.1	0.3 ± 0.7	0.0050*
Reduced need for sleep (ASRM)	0.8 ± 1.3	0.4 ± 0.9	0.0084*
Increased activity (ASRM)	0.3 ± 0.9	0.1 ± 0.5	0.0274
Multiple logistic regression analysis (overlapping symptoms between PTSD and bipolar disorder): Chi-square = 37.73; df = 5; *P* < 0.0001			
Predictors of PTSD	OR (95% CI)		
Bipolar spectrum score	1.27 (1.05–1.54)		
Irritability	2.07 (1.14–3.76)		
Difficulty in falling asleep	1.26 (1.03–1.54)		
Middle nocturnal insomnia	1.28 (1.02–1.61)		
Poor concentration	1.34 (0.98–1.87)		
Multiple logistic regression analysis: Chi-square = 55.61; df = 9; *P* < 0.0001			
Predictors of PTSD	OR, (95% CI)		
Bipolar spectrum score	1.21 (0.99–1.48)		
Irritability	2.07 (1.12–3.81)		
Difficulty in falling asleep	1.26 (1.02–1.55)		
Middle nocturnal insomnia	1.27 (1.01–1.61)		
Poor concentration	1.23 (0.89–1.70)		
Childhood parental neglect	1.42 (0.72–2.83)		
Childhood emotional abuse	1.35 (0.64–1.82)		
Childhood physical abuse	1.95 (1.05–3.63)		
Childhood sexual abuse	0.73 (0.41–1.29)		

ASRM, Altman Self-Rating Mania Scale; CAST, Concise Associated Symptoms Tracking; CI, confidence interval; OR, odds ratio; PTSD, post-traumatic stress disorder; PDSQ, Psychiatric Diagnostic Screening Questionnaire.

### Childhood maltreatment exposure and bipolar spectrum

Our research sample more often reported childhood exposure to emotional abuse (*N* = 212; 44%) and parental neglect (*N* = 194; 40%), whereas physical (*N* = 109; 23%) and sexual (*N* = 115; 24%) abuse were less common. All CMT subtypes, except for physical abuse (*t* = 1.71; *P* = 0.0875), were associated with higher bipolar spectrum scores at univariate level (emotional abuse: *t* = 4.32; *P* < 0.0001; neglect: *t* = 3.74; *P* = 0.0002; sexual abuse: *t* = 2.45; *P* = 0.0146). Multiple regression analysis, however, identified childhood emotional abuse as the only independent predictor of the bipolar spectrum (beta = 0.216; *t* = 3.90; *P* = 0.0001) (Table 3).

**Table 3:** Childhood maltreatment and bipolar spectrum score^a^

	Present		Absent		*P*
	*N*	Mean ± SD	*N*	Mean ± SD	
Childhood parental neglect	144	1.3 ± 1.2	288	0.9 ± 1.0	0.0002*
Childhood emotional abuse	212	1.3 ± 1.2	270	0.8 ± 1.0	<0.0001*
Childhood physical abuse	109	1.2 ± 1.2	373	1.0 ± 1.1	0.0875
Childhood sexual abuse	115	1.2 ± 1.1	367	1.0 ± 1.1	0.0184*
Multiple regression: *F* = 7.37; df = 3; *P* = 0.0001					
Childhood emotional abuse	Beta = 0.216	*t* = 3.89	*P* < 0.001*		
Childhood physical abuse	Beta = −0.073	*t* = 1.29	*P* = 0.1974		
Childhood sexual abuse	Beta = 0.066	*t* = 1.35	*P* = 0.1775		

a Bipolar spectrum score: age of onset <21 years (1 point) + subthreshold hypomania (2 points) + DMX (2 points).

DMX, depressive mixed state (see manuscript).

### Antidepressant treatment outcome and mood activation switch

A total of 395 patients (82%) completed 6 week period of antidepressant use. Of them, 183 subjects (46%) were classified as responders and 117 (30%) achieved remission. None of the two outcome definitions was associated with PTSD comorbidity (Response: PTSD = 35/80 no PTSD = 148/315; χ^2^ = 0.268; *P* = 0.605; Remission: PTSD = 18/80 no PTSD = 99/315; χ^2^ = 2.391; *P* = 0.122), neither after controlling for depression severity (MLR χ^2^ = 8.243; *P* = 0.0162; remission: OR, 0.41–1.32). Instead, patients with PTSD comorbidity showed higher levels of residual suicide propensity (CHRT) than their counterparts without PTSD (*P* = 0.0092), similar to individuals who reported histories of parental neglect (*P* = 0.0053) and emotional abuse (*P* = 0.0039) during childhood (Table 4). However, after controlling for baseline CHRT scores, only PTSD comorbidity was confirmed to be correlated with suicide propensity at week 6 (multiple regression: *F* = 36.51; *P* < 0.0001; PTSD: beta = 0.107; *P* = 0.0218) (Table 4). Mood activation was found to occur in 204 out of 425 subjects (48%) who had been receiving antidepressant treatments for at least 14 days. The mood activation group included more cases with subthreshold hypomania (χ^2^: 6.50; df = 1; *P* = 0.011) and childhood emotional abuse (χ^2^: 5.27  df = 1; *P* = 0.0217) in comparison with patients who did not switch their mood (Table 5). MLR analysis results displayed that subthreshold hypomania (OR, 1.04–4.09), DMX (OR, 1.00–4.19) and childhood emotional abuse (OR, 1.01–2.22) were independently associated with mood activation risk (Table 5).

**Table 4:** Antidepressant treatment outcome (week 6) (*N* = 395

	With PTSD*N* = 80	Without PTSD*N* = 315	*P*
Response	35 (0.44)	148 (0.47)	0.6040
Remission	18 (0.22)	99 (0.31)	0.1220
Suicide propensity	11.15 ± 8.55	8.59 ± 7.54	0.0092*
Suicide ideation	1.41 ± 2.26	1.03 ± 1.81	0.1669
	Neglect *N* = 152	No neglect *N* = 241	*P*
Suicide propensity	10.47 ± 8.10	8.23 ± 7.50	0.0053*
Suicide ideation	1.31 ± 2.10	0.98 ±1.77	0.1049
	Emotional abuse *N* = 168	No abuse *N* = 225	*P*
Suicide propensity	10.43 ± 8.33	8.10 ± 7.25	0.0039*
Suicide ideation	1.38 ± 2.25	0.90 ± 1.58	0.0182*
	Physical abuse *N* = 84	No abuse *N* = 311	*P*
Suicide propensity	10.55 ± 7.71	8.70 ± 7.80	0.0546
Suicide ideation	1.44 ± 2.15	1.01 ± 1.84	0.0982
	Sexual abuse *N* = 93	No abuse *N* = 302	*P*
Suicide propensity	10.44 ± 7.64	8.68 ± 7.82	0.0572
Suicide ideation	1.22 ± 1.89	1.07 ± 1.92	0.5233
Multiple regression:	*F* = 36.51	df = 5	<0.0001
Dependent variable:	Predictors: emotional abuse is excluded (weakest predictor)		
Suicide propensity (week 6)	Suicide probensity (baseline)	Beta = 0.558	<0.0001*
	PTSD	Beta = 0.108	0.0207*
	Neglect	Beta = 0.024	0.6528
	Physical abuse	Beta = 0.106	0.0504
	Sexual abuse	Beta = 0.027	0.5829

Role of PTSD and childhood maltreatment. CMT: parental neglect; sexual abuse; physical abuse; emotional abuse.

CMT, childhood maltreatment; PTSD, post-traumatic stress disorder.

**Table 5:** Predictors of mood activation (ASRM ± 6) switch after at least 14 days of treatment (*N* = 425 patients)

	Switch*N* = 204	No switch*N* = 221	*P*
Deperessive mixed state (DMX) (*N* = 37)	24 (0.12)	13 (0.06)	0.0308
Subthreshold hypomania (*N* = 42)	28 (0.14)	14 (0.06)	0.0103*
PTSD (*N* = 85)	46 (0.22)	39 (0.18)	0.2070
Childhood parental neglect (*N* = 169)	89 (0.44)	80 (0.36)	0.1179
Childhood emotional abuse (*N* = 186)	101 (0.49)	85 (0.38)	0.0217*
Childhood physical abuse (*N* = 94)	50 (0.24)	44 (0.19)	0.2538
Childhood sexual abuse (*N* = 104)	52 (0.25)	52 (0.23)	0.6386
Multiple logistic regression (tested predictors with univariate *P* ≤ 0.1): Chi-square: 14.50 df: 3; *P* = 0.0023			
	OR (95% CI)		
Depressive mixed state (DMX):	2.05 (1.00–4.19)		
Subthreshold hypomania	2.06 (1.04–4.09)		
Childhood emotional abuse	1.45 (1.01–2.22)		

ASRM, Altman Self-Rating Mania Scale (Altman, 1998); CI, confidence interval; OR, odds ratio; PTSD, post-traumatic stress disorder.

## Discussion

In our sample, about one in five patients had PTSD comorbidity. This figure was distant from 50 to 70% reported in Veteran outpatients (Zisook *et al.*, 2016; Mohamed *et al.*, 2020) and socioeconomically disadvantaged groups (Grote *et al.*, 2016), but substantially similar to 33–36% displayed in other clinical samples (Green *et al.*, 2006; Campbell *et al.*, 2007). Instead, the prevalence of PTSD was significantly lower in the Sequenced Treatment Alternatives to Relieve Depression study (STAR*D), which only identified 122 cases from 2280 participants (5%) (Steiner *et al.*, 2017). Such a difference was not related to PTSD assessment, which was conducted via PDSQ administration as well, but it could reflect the larger proportion of patients (54%) in our sample who were victims of maltreatment during childhood and, consequently, exposed to traumatization.

### Diagnostic role of post-traumatic stress disorder and childhood emotional abuse as markers of subthreshold bipolarity

A clear-cut result of our study was to associate PTSD and childhood emotional abuse with a variety of bipolar validators assessed lifetime (Angst *et al.*, 2003; Benazzi, 2009; Zimmermann *et al.*, 2009; Mazzarini *et al.*, 2018) and during a single MDE (Benazzi, 2001, 2005, 2008; Akiskal *et al.*, 2005; Perugi *et al.*, 2015). These findings are largely consistent with epidemiological data that suggest high levels of diagnostic comorbidity between PTSD and bipolar disorder (Otto *et al.*, 2004; Graves *et al.*, 2007; Neria *et al.*, 2008; Hernandez *et al.*, 2013; McLay *et al.*, 2014). Moreover, prior to ours, other studies have displayed a correlation between childhood adversity and bipolar features in major depressed patients (Park, 2017). It is plausible that childhood traumas and maltreatment are not simply more widespread among subjects with bipolar disorders (Janiri *et al.*, 2015; Palmier-Claus *et al.*, 2016) but, rather, risk factors for bipolar illness (Quide et al., 2020). The comorbidity between PTSD and bipolar disorder could be explained by the overlap of some symptoms between these conditions. In particular sleep disturbance, difficulty concentrating, increased risk-taking behavior and irritability are often reported in patients with PTSD and bipolar disorders (Cogan *et al.*, 2021) and they also represent the typical profile of mixed depression (Perugi *et al.*, 2015; Brancati *et al.*, 2019). Therefore a valuable result of this study was to demonstrate that control for irritability, insomnia and poor concentration did not modify the association between bipolar risk score and PTSD. This would suggest that the co-occurrence of PTSD and bipolar spectrum disorder might be true comorbidity rather than a mere artifact, although there is a need for further studies to corroborate this hypothesis. However, if experiences of CMT were added to irritability and insomnia, the association between bipolar risk score and PTSD was no more significant. Overall these results seem to indicate that the association between bipolar spectrum and PTSD could be at least in part mediated by CMT. Nevertheless, further studies are necessary to ascertain the effectiveness of assessing PTSD symptoms and childhood maltreatment in patients with DSM unipolar depression in order to improve the identification of bipolar spectrum disorders.

### Prognostic impact of post-traumatic stress disorder and childhood emotional abuse

We found that the presence of PTSD was associated with more severe depressive symptoms, notably higher negative self-outlook, anxiety and insomnia, work and social impairment and increased suicidal tendency. This picture was consistent with prior studies suggesting that subjects with comorbid major depression and PTSD might have more psychopathological manifestations as well as a higher suicide risk than those with either condition alone (Morina *et al.*, 2013). Moreover, our findings mirrored those emerging from the STAR*D study, which displayed the impact of PTSD comorbidity on depression severity and functional impairment as well (Steiner *et al.*, 2017). As baseline clinical severity was found to negatively affect antidepressant treatment outcomes (Friedman *et al.*, 2012), we expected that PTSD comorbidity was associated with a less favorable antidepressant response. Such a correlation had been documented in STAR*D sample (Steiner *et al.*, 2017). Conversely, we failed to demonstrate any association between PTSD and antidepressant-related outcomes. This result was in line with a recent study, based on CO-MED data like the present one, in which exposure to CMT had no impact on antidepressant response (Medeiros *et al.*, 2021). Nevertheless, PTSD might exert a negative prognostic influence on major depression. Indeed, in our sample, PTSD group was characterized by higher levels of the residual propensity for suicidal behavior after acute antidepressant treatment and this association was confirmed even accounting for the higher degree of suicidality reported at baseline.

Another outcome variable analyzed in the current study was the new onset of mood activation symptoms during antidepressant use. The occurrence of hypomanic symptoms within a depressive episode of unipolar disorder was already investigated using CO-MED sample (Jha *et al.*, 2018). In that study, patients who endorsed hypomanic symptomatology were characterized by lower remission rates with escitalopram monotherapy and venlafaxine plus mirtazapine combination. Instead, our results corroborated the role of intra-MDE hypomanic symptoms and sub-threshold hypomanic episodes in predicting mood switch during antidepressant therapy. Interestingly, a similar correlation with mood activation was shown by childhood emotional abuse. These findings are intriguing and provide new evidence about predictors of mood activation in apparently unipolar depression (DelBello *et al.*, 2003; Celik *et al*., 2016).

### Stregth and limitations

This study analyzed in detail depressive symptomatology and bipolar features using both cross-sectional and longitudinal approaches. Axis I comorbidities were also carefully assessed. Hence, it was possible to disentangle the effect of several potential confounding variables while investigating the association between PTSD comorbidity and bipolar spectrum. The onset of mood activation during antidepressant treatment is a concern for clinicians and our study provided some cues to predict this risk. On the other hand, the main limitations are related to the post hoc nature of our analysis and to the retrospective approach used to investigate a large number of variables (e.g. onset of the first depressive episode; lifetime occurrence of subthreshold hypomania; CMT, etc). Moreover, common symptoms of mixed depression such as thought racing, distractibility or reckless activity could not be assessed using the ASMR scale, thus the prevalence of DMX could have been underestimated as well as some reliable bipolar validators (e.g. family history) were not available. Finally, there is evidence that the effects of childhood emotional abuse on suicidality are at least in part mediated by emotional abuse and depressive symptoms experienced in adulthood (Lee, 2015). Therefore, a caveat of this study was that it could analyze emotional abuse during childhood but not re-victimization in adult age.

### Conclusion

Maltreatment experiences occurring in childhood and PTSD symptoms in adult age are commonly reported by subjects with MDD. Their presence is associated with severe forms of depression and, as suggested by current data, they might be putative markers of bipolar spectrum. Therefore, PTSD symptoms and CMT should be carefully assessed in all patients who endorse an MDE before choosing pharmacological treatment, in order to minimize risk for a mood activation switch.

## Acknowledgements

We thank the NIMH for having had the possibility of analyzing their data on the COMED sample. We also thank the authors of previous publications in this dataset, and foremost, we thank the subjects and their families who accepted to be enrolled in the study. Data and biomaterials were obtained from the limited access datasets distributed from the NIH. The ClinicalTrials.gov identifier is NCT00590863.

Data were obtained for analysis from the National Institute of Mental Health, Bethesda, Maryland, US (Request ID 5ce26a95712d8). The CO-MED trial was conducted according to the Principles of the Helsinki Declaration. The study protocol was reviewed and approved by ethical committees at local recruitment sites. All subjects selected by clinicians were included in the screening phase after obtaining their written informed consent. This research group certifies that data collected for the CO-MED trial were exclusively used for scientific investigation. Before obtaining access to data, the objectives of our investigation were clearly described in the request form.

A.S. and P.O. conceived the study; P.O. performed the analyses and drafted the manuscript; A.S. revised and interpreted the results, discussed the findings and drafted the final version of the article.

## Conflicts of interest

A.S. is or has been a consultant to or has received honoraria or grants unrelated to the present work from Abbott, Abbvie, Angelini, Astra Zeneca, Clinical Data, Boheringer, Bristol Myers Squibb, Eli Lilly, GlaxoSmithKline, Innovapharma, Italfarmaco, Janssen, Lundbeck, Naurex, Pfizer, Polifarma, Sanofi, Servier and Taliaz. P.O. has no conflicts of interest.
